# Assessment of Maximal Aerobic Capacity in Ski Mountaineering: A Laboratory-Based Study

**DOI:** 10.3390/ijerph18137002

**Published:** 2021-06-30

**Authors:** Verena Menz, Martin Niedermeier, Rainer Stehle, Hendrik Mugele, Martin Faulhaber

**Affiliations:** Department of Sport Science, University of Innsbruck, 6020 Innsbruck, Austria; martin.niedermeier@uibk.ac.at (M.N.); rainer.stehle93@gmail.com (R.S.); hendrik.mugele@uibk.ac.at (H.M.); martin.faulhaber@uibk.ac.at (M.F.)

**Keywords:** ski mountaineering, sport-specific exercise test, maximum oxygen consumption, performance, SKIMO

## Abstract

This study aims to evaluate the agreement in maximum oxygen consumption ($\dot{V}$O_2_max) between a running protocol and a ski mountaineering (SKIMO) protocol. Eighteen (eleven males, seven females) ski mountaineers (age: 25 ± 3 years) participated in the study. $\dot{V}$O_2_max, maximum heart rate (HRmax), and maximum blood lactate concentration (BLAmax) were determined in an incremental uphill running test and an incremental SKIMO-equipment-specific test. $\dot{V}$O_2_max did not differ between the SKIMO and uphill running protocols (*p* = 0.927; mean difference –0.07 ± 3.3 mL/min/kg), nor did HRmax (*p* = 0.587, mean difference –0.7 ± 5.1 bpm). A significant correlation was found between $\dot{V}$O_2_max SKIMO and $\dot{V}$O_2_max running (*p* ≤ 0.001; ICC = 0.862 (95% CI: 0.670−0.946)). The coefficient of variation was 4.4% (95% CI: 3.3−6.5). BLAmax was significantly lower for SKIMO compared to running (12.0 ± 14.1%; *p* = 0.002). This study demonstrates that $\dot{V}$O_2_max determined with a traditional uphill running protocol demonstrates good agreement with an equipment-specific SKIMO protocol.

## 1. Introduction

In the European Alps and other alpine countries, ski mountaineering (SKIMO) has developed into a fast-growing winter sport and leisure activity [1]. Several national and international competitions, including World Cups and Youth Olympic Games, have been arranged in SKIMO (ISMF 2019; www.ismf-ski.org, accessed on 4 June 2019). SKIMO competitions contain three major disciplines: single race, team race, and vertical race. Typically, SKIMO single races last between 1.5 and 2.5 h for the fastest racers, where most of the time (>80% of total race time) is spent on the ascent [2]. SKIMO competitions are usually held at altitudes around 2000 m above sea level and have been recognized as one of the most demanding endurance disciplines [2,3]. The high physiological demand is mirrored in exercise intensity during SKIMO competitions, which has been reported to be close to the respiratory compensation point [2,4]. 

Despite its growing popularity, only a few studies have been carried out on the physiological aspects of SKIMO. The energy cost of SKIMO was investigated by few studies [2,5,6,7], demonstrating that SKIMO is more energy demanding than cross-country skiing or snowshoe walking [2]. This is partly due to the extensive equipment (8 ± 2 kg) [1] necessary for SKIMO [3]. Moreover, SKIMO racing performance was reported to significantly correlate with maximum oxygen uptake ($\dot{V}$O_2_max), body mass of the athlete [3], first ventilatory threshold (VT1), and respiratory compensation threshold (RCT) [2]. As demonstrated in previous studies, factors such as exercise modality and test protocol, including test duration and stage length, significantly affect the $\dot{V}$O_2_max attained during an exercise test [8,9,10]. Indeed, the concept that athletes should be tested sport-specifically is supported by the results of Pinna et al. [11]. They demonstrated that predicting $\dot{V}$O_2_max in trained swimmers from non-specific exercise tests such as cycling or arm cranking does not provide data similar to those obtained from swimming.

Therefore, traditional laboratory $\dot{V}$O_2_max tests (e.g., cycling, level grade treadmill running) may provide less precise information on the physiological demands of exercising with SKIMO equipment when differences between SKIMO and traditional $\dot{V}$O_2_max tests are considered. Specifically, SKIMO involves uphill moving, the use of more muscle mass due to the active involvement of both the trunk and upper body [2], as well as the additional equipment carried [3], compared to traditional $\dot{V}$O_2_max tests. Hence, as SKIMO places these specific physiological demands, athletes might benefit from a SKIMO-equipment-specific $\dot{V}$O_2_max test in terms of robust training planning and performance diagnosis. However, a SKIMO-equipment-specific $\dot{V}$O_2_max test needs to fulfill the criterion of being a valid test. While several studies [3,4,5] have used traditional but unspecific running exercises to assess $\dot{V}$O_2_max in ski mountaineers, others [2,12] have evaluated $\dot{V}$O_2_max in an incremental SKIMO field test on a groomed snowy Alpine track using acoustic signals to match the required speed. Another approach in a more controlled laboratory environment is the use of roller skis [6,13,14] on large motorized roller-skiing treadmills, which most closely resembles SKIMO exercise. However, the friction coefficient between roller skis and SKIMO skis (fitted with adhesive skins) and, in particular, the dimensions of the skis are different, which limits the use of roller skis in terms of a sport-specific context. To the best of our knowledge, to date, only one study has used a SKIMO-specific incremental step protocol to evaluate $\dot{V}$O_2_max in eight elite SKIMO athletes [15] in a laboratory environment and compared it to a standardized cycle test, exhibiting inconsistency between cycling and SKIMO $\dot{V}$O_2_max. 

As existing methods to assess $\dot{V}$O_2_max in SKIMO are non-sport- and equipment-specific, complex to conduct, or do not take into account muscle activity for uphill movements, the present study aims at evaluating a standardized maximal ramp protocol for laboratory SKIMO testing on a commercially available treadmill. Hence, the overall aim of the study is to evaluate the agreement in $\dot{V}$O_2_max between a running protocol and a SKIMO-specific protocol. 

## 2. Materials and Methods

### 2.1. Participants

Study participants were recruited via personal contacts and social media between March and April 2019. Eighteen healthy (eleven males, seven females) and experienced ski-mountaineers (34 ± 15 SKIMO tours per season) were included in the study. Prior to the first exercise test (either running or SKIMO), participants underwent routine pre-participation screening by answering an adapted physical activity readiness questionnaire (PAR-Q) [16]. Exclusion criteria were pre-existing acute or chronic diseases, pregnancy, and lactation period. Before providing their verbal and written informed consent to participate in the study, participants were provided detailed information about the procedure and potential risks of the study. The study met the ethical standards set by the Declaration of Helsinki, and the procedures of the study were approved by the local Board for Ethical Questions in Science. A sensitivity analysis for the present sample was conducted using G*Power 3.1 (University of Düsseldorf, Düsseldorf, Germany). Based on the assumptions of alpha = 0.05, power = 0.80, nonsphericity correction = 1, and r among repeated measures = 0.5 and using a repeated-measures ANOVA as the statistical analysis, an effect size of partial eta^2 > 0.11 was revealed as significant with the present sample size of 18 participants. Participants’ demographic and anthropometric characteristics are shown in Table 1. 

### 2.2. Design

This randomized crossover study consisted of an incremental uphill running test and an incremental SKIMO-equipment-specific test, separated by at least seven days (maximum 14 days). Participants were advised to refrain from intense exercise and alcohol 24 h before each exercise test.

### 2.3. Exercise Testing

The running test was conducted on a conveyer belt treadmill (h/p/cosmos pulsar®, h/p/cosmos Sports and Medical, Nussdorf, Germany). The participants wore a harness attached to the safety arch to prevent potential falls. The SKIMO test was performed on a treadmill with a slat belt surface (Woodway, Waukesha, WI, USA) without a safety arch. To provide adequate safety for the participants, two assistants spotted the participants in the last few stages of the test. 

Cardiorespiratory parameters were measured continuously using an open spirometric system (Oxycon mobile, CareFusion, Baesweiler, Germany) that was calibrated according to the manufacturer’s guidelines before each test. First and second ventilatory thresholds (VT1 and VT2, respectively) were later determined by visual inspection from two experienced researchers. For determining VT1, the V-slope plot ($\dot{V}$CO_2_ vs. $\dot{V}$O_2_) as well as the increase in $\dot{V}$E/$\dot{V}$O_2_, with no concomitant increase in $\dot{V}$E/$\dot{V}$CO_2_, were considered for evaluation. For determining VT2, the second disproportional increase in $\dot{V}$E vs. $\dot{V}$CO_2_ and the increase in $\dot{V}$E/$\dot{V}$CO_2_ were visually inspected. Heart rate (HR) was determined by a chest belt (Wear Link, Polar, Kempele, Finland) and transmitted to the spirometric device. 

The non-SKIMO-equipment-specific exercise test was an uphill running protocol that was previously used in several studies in trained participants [17,18], described in detail in Table 2. Briefly, exercise started at 5.0 km/h and 5% inclination for two minutes; then, the inclination was set at 10% for another two minutes. Subsequently, the speed was increased to 6.0 km/h, and inclination was augmented by 2% every minute until 20%. Then, the running speed was increased by 1.0 km/h per minute while the inclination was kept constant at 20%. The SKIMO protocol was designed in conformity with the previously described uphill running protocol and preceding SKIMO studies [2,12,15]. Starting at 3.0 km/h and an inclination of 10%, the inclination was increased after two minutes to 20% for another two minutes. Then, speed was augmented to 3.5 km/h at an inclination of 20%. Hereafter, speed was kept constant at 3.5 km/h, whereas inclination was increased each minute by 2% until 30% was reached. Finally, speed was increased by 0.5 km/h each minute, while inclination was kept at 30% (Table 3). Tests were completed when participants reached volitional exhaustion. A test was considered maximal when three of the following criteria were fulfilled: (1) $\dot{V}$O_2_peak plateau at peak exercise; (2) respiratory exchange ratio ≥ 1.10; (3) peak HR ≥ 90% of the theoretical maximal HR (220—age); (4) indication of maximal exhaustion by the athlete [19]. Maximum oxygen consumption was defined as the highest 30-second average during the test. Directly after terminating the treadmill test, a capillary blood sample was collected from the earlobe to assess the maximal blood lactate concentration (BLAmax; Biosen C line, EKF Diagnostics, Barleben, Germany), and the ratings of perceived exertion (RPEmax; separately for breathing and lower limb muscles) according to the Borg scale [20] were recorded. Female and male participants performed the SKIMO test on 158 and 174 cm SKIMO skis, respectively (Tour 88 Ski, Dynafit, Aschheim, Germany). The skis were equipped with SKIMO skins (Speed Fell Tour 88, Dynafit, Aschheim, Germany) and SKIMO bindings (ST Radical, Dynafit, Aschheim, Germany) on the medium heel raiser. Participants wore a sex-specific SKIMO boot model (HOJI PX W and HOJI PX, Dynafit, Aschheim, Germany, for females and males, respectively) in their individual shoe size. Moreover, extendable ski poles equipped with rubber stoppers, adjusted to individual body heights, were used for the test, resulting in a total added SKIMO gear weight of 3900 g for female participants and 4200 g for male participants. 

### 2.4. Statistical Analyses

Statistical analyses were conducted using IBM SPSS Statistics for Windows, version 25 (IBM Corp., Armonk, NY, USA). Values are presented as mean ± SD. The data were tested for normal distribution with the Shapiro–Wilk test. The primary outcome parameter was the attained $\dot{V}$O_2_max (mL/min/kg) during the running and SKIMO exercise tests. The identical units in both conditions allowed a reliability approach, following Hopkins [21] and Weir [22]. A repeated-measures analysis of variance (ANOVA) with one within-subject factor (type of test: running, SKIMO) was used to determine the differences between running and SKIMO exercise tests. In addition, an intraclass correlation coefficient (ICC(3,1); two-way mixed consistency) was calculated between running and SKIMO $\dot{V}$O_2_max [22]. The typical error (TE), including 95% confidence intervals (95% CIs), were calculated using the standard deviation of differences between running and SKIMO divided by √2 [21]. The coefficient of variation (CV), including 95% confidence intervals, were calculated using the TE divided by the average $\dot{V}$O_2_max (running; SKIMO) multiplied by 100 [21].

Both a Bland-Altman plot and a scatterplot of $\dot{V}$O_2_max (running versus SKIMO) were created. The Bland-Altman plot consisted of the difference between $\dot{V}$O_2_max running and SKIMO and the average $\dot{V}$O_2_max (running; SKIMO), including 95% confidence intervals of the average difference [23]. The 95% confidence intervals were referred to as the limits of agreement. A simple linear regression analysis was conducted with average $\dot{V}$O_2_max as the independent variable and difference in $\dot{V}$O_2_max as the dependent variable to analyze proportional bias (e.g., higher measurement error in higher $\dot{V}$O_2_max values). *p*-values < 0.05 (two-tailed) were considered to indicate statistical significance. 

## 3. Results

All participants fulfilled the criteria for a maximal test according to Cunha et al. [19] for the running test, and all but one participant (fulfilled only two criteria) fulfilled the criteria for the SKIMO test. No harmful incident was observed during all tests.

**Figure 1:**
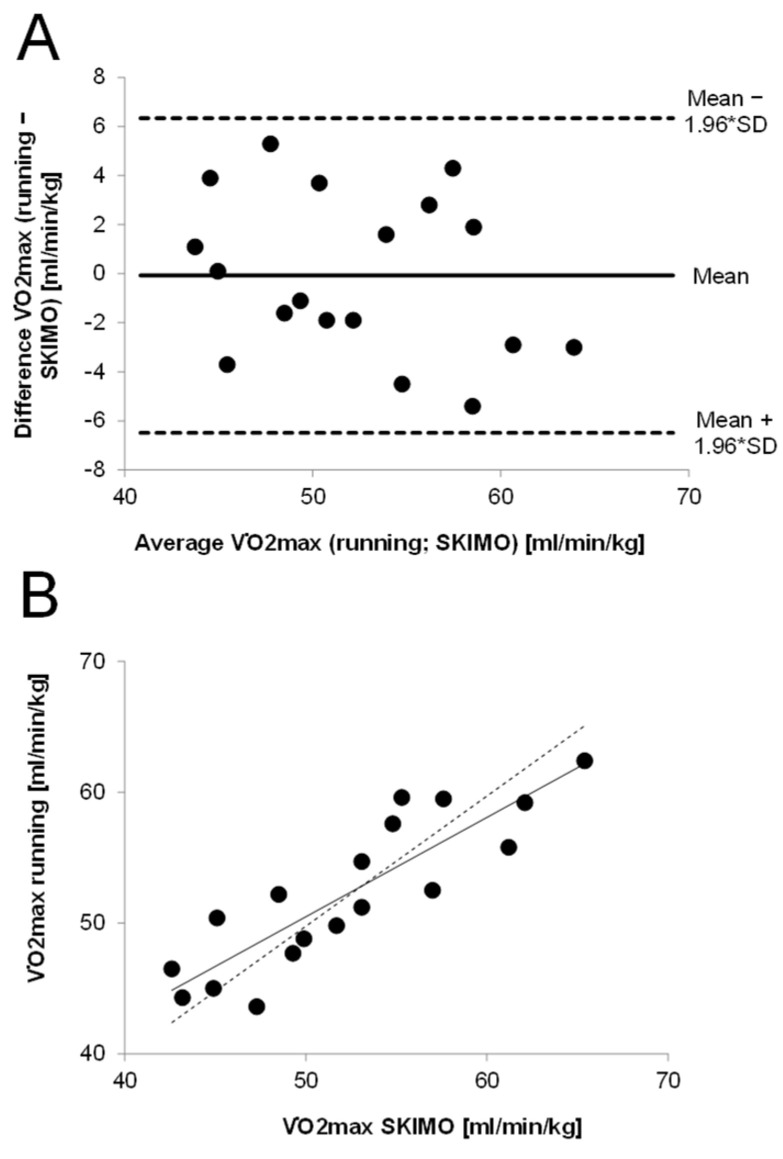
Agreement in $\dot{V}$O_2_max values between running and SKIMO. (**A**): Bland–Altman plot showing the differences between running and SKIMO against average $\dot{V}$O_2_max values. (**B**): $\dot{V}$O_2_max values for uphill running against SKIMO, including regression line (solid) and ideal line (dashed).

Maximum oxygen uptake (mL/min/kg) values obtained during the running test did not significantly differ from the SKIMO-equipment-specific test. There was a significant correlation between SKIMO and running for $\dot{V}$O_2_max (mL/min/kg) (*p* < 0.001; ICC [95% CI] = 0.862 [0.670−0.946]). The TE for the relative $\dot{V}$O_2_max was 2.3 mL/min/kg [95% CI: 1.7−3.4] and for the absolute $\dot{V}$O_2_max, 164 mL/min [95% CI: 123−241]. The coefficient of variation (CV) was 4.4% [95% CI: 3.3−6.5] for relative and absolute $\dot{V}$O_2_max values. The Bland–Altman plot shows that all values were within the limits of agreement (Figure 1). 

The mean difference was –0.07 mL/kg/min, indicating that SKIMO and running $\dot{V}$O_2_max values were largely similar. The limits of agreement ranged between −6.5 and 6.3 ml/min/kg. The plot does not indicate that the difference in $\dot{V}$O_2_max is dependent on average $\dot{V}$O_2_max, which was confirmed by a non-significant linear regression analysis (β = −0.27, *p* = 0.282). The time to exhaustion (TTE) was significantly higher for running compared to SKIMO (15.0 ± 4.9%, *p* ≤ 0.001), whereas BLAmax was significantly higher in running compared to SKIMO (12.0 ± 14.1%; *p* = 0.002). Other mean maximal and threshold values are presented in Table 4.

## 4. Discussion

The main purpose of this study was to compare the $\dot{V}$O_2_max values obtained during an uphill running test and a SKIMO-equipment-specific test in a laboratory setting. It was demonstrated that the $\dot{V}$O_2_max values of uphill running and SKIMO showed acceptable indices of agreement, including a high correlation of ICC > 0.8, a low mean difference of −0.07 mL/min/kg, and a low CV of 4.4%. However, BLAmax and TTE significantly differed between both protocols. 

The participants in the present study elicited a mean $\dot{V}$O_2_max of 52.3 ± 5.8 and 52.3 ± 6.6 mL/min/kg for running and SKIMO, respectively. The Bland–Altman plot shows that 100% were within the limits of agreement, exhibiting no outliers and symmetry in distribution (Figure 1). The mean difference between both tests was −0.07 mL/min/kg, showing a negligible systematic bias and indicating that SKIMO and running $\dot{V}$O_2_max values were largely similar. The mean difference of the present study is comparable to test–retest differences, where a mean difference of −0.04 mL/min/kg was reported [24]. However, the limits of agreement calculated in the present study (−6.5 and 6.3 mL/min/kg) were relatively large compared to previously reported values of −2.04 and 1.96 mL/min/kg [24]. Admittedly, Loe et al. [24] assessed test–retest reliability and tested more than 3000 participants. Since the present study had a sample size of only 18 participants, this may have resulted in a higher standard deviation and, accordingly, larger limits of agreement. However, based on the limits of agreement in the present study, a person with a running $\dot{V}$O_2_max of, e.g., 50 mL/min/kg in the worst case, would exhibit a SKIMO $\dot{V}$O_2_max as high as 56.3 mL/min/kg or as low as 43.5 mL/min/kg [25]. The present study revealed a CV of 4.4% and a TE of 2.3 mL/min/kg for $\dot{V}$O_2_max. Considering that the CV for test–retest $\dot{V}$O_2_max measurements is reported to be around 5% [26], the CV of the present study lies within this magnitude, substantiating that the variation in $\dot{V}$O_2_max values is low between running and SKIMO.

To the best of our knowledge, there is only one study that has previously compared a SKIMO-specific exercise test with a traditional $\dot{V}$O_2_max test [15]. Although not statistically different, Schöffl et al. [15] reported notable differences between the $\dot{V}$O_2_max values attained in SKIMO and cycling tests. On average, participants achieved a higher $\dot{V}$O_2_max on the bike compared to SKIMO (62.1 ± 9.7 and 56.8 ± 11.9 mL/min/kg, respectively), resulting in a mean difference of −5.3 mL/min/kg, whereas the present study exhibited a mean difference of only −0.07 mL/min/kg. The disparity in the results might have several reasons. First, Schöffl et al. [15] used a discontinuous SKIMO protocol but a continuous cycling protocol with a stage duration of three minutes, while the present study used a continuous ramp protocol with a stage duration of one minute (after the four-minute warm-up period) for both uphill running and SKIMO. Research has indicated that incremental ramp tests lasting eight to 12 min will result in higher $\dot{V}$O_2_max values than prolonged incremental tests [27,28]. Second, while in the present study, SKIMO was compared to uphill running, which rather resembles SKIMO due to the utilized muscle mass, Schöffl et al. [15] compared it to cycling exercise, where less muscle mass is utilized. Therefore, it is somewhat surprising that the participants, who were members of the German national SKIMO team, exhibited lower $\dot{V}$O_2_max values in the SKIMO test compared to the bike test. Despite the fact that the authors stated that cardiorespiratory exhaustion was reached in all test protocols and that it was feasible for athletes, it can only be speculated that the SKIMO athletes could not have reached maximum exhaustion during the SKIMO test, probably due to the high speed required for exhaustion and the coordinative problems associated with the high treadmill speed. 

Nevertheless, the present study reveals that not all measured physiological responses of SKIMO-equipment-specific exercise test are consistent with the uphill running test, as the SKIMO protocol generated less accumulation of BLAmax compared to the uphill running protocol. This may indicate different metabolic demand and substrate utilization. It might be speculated that the activation of the lower extremity muscle volume, which has been reported to be higher in uphill running compared to level running [29], may be responsible for the difference in BLAmax. This may be supported by the fact that RPEmax for the lower extremity tends to be higher in running compared to SKIMO. Although slightly higher, the same pattern was reported in the data set of Schöffl et al. [15], demonstrating higher BLAmax on the bike compared to SKIMO. Surprisingly, in the present study, neither $\dot{V}$O_2_ nor HR at VT2 and HRmax differed between both protocols, which would have been of practical relevance when considering threshold-based or %HRmax-based training prescriptions. This questions the further benefit of a SKIMO-equipment-specific exercise test, which is much more material-intensive than traditional treadmill protocol, eliciting the same test results. Furthermore, TTE differed between both protocols, with the SKIMO protocol lasting 108 ± 30 s longer. The optimal test duration for $\dot{V}$O_2_max testing is supposed to be between 8 to 12 min [28,30]. The running test lasted, on average, approximately 12 min, whereas the SKIMO test lasted almost 14 min. However, when taking into account that the warm-up (first two stages in the protocol) was included in both protocols, SKIMO TTE still lies within the ideal duration and is comparable to a reported field test duration with a similar protocol [2]. 

Exercising with SKIMO equipment on a motorized treadmill is demanding in terms of coordination. A certain amount of coordinative SKIMO skills is absolutely required to guarantee a safe realization of the test and is a basic requirement for maximal exertion. No incidents occurred during the SKIMO exercise in the present study with recreationally active ski mountaineers. Due to technical and infrastructural conditions, participants did not wear a safety harness during the SKIMO test. However, to ensure their safety, two assistants were standing on each side of the treadmill, spotting the participant. The best participants reached a maximum speed of 6.5 km/h at an inclination of 30% (16.7°). Consequently, treadmill speeds up to 6.5 km/h may be considered safe in recreationally active ski mountaineers. In SKIMO athletes, Schöffl et al. [15] reported a maximum treadmill speed of 8.0 km/h with an inclination of 36.4% (20°). However, this value is still below that reported in SKIMO races, where the athletes can reach a maximum uphill speed of 10.5 ± 1.3 km/h [2]. 

The maximum inclination was restricted to 30% because the SKIMO protocol was conducted on a treadmill traditionally built for running. In turn, this might have limited the test procedure since it may be less challenging to exercise at a higher inclination at lower speeds. However, most laboratories are equipped with standard running treadmills with a maximal inclination of 30%, and, thus, it is rather useful to have equipment-suited protocols. Another limiting factor that has to be considered is the fact that we did not compare the laboratory SKIMO results with SKIMO performance in the field. In the present study, aerobic capacity was measured 560 m above sea level, whereas SKIMO is usually performed at altitudes higher than that. Wehrlin and Hallén [31] reported a 6.3% decrease in $\dot{V}$O_2_max per 1000 m increasing altitude. The decline in aerobic capacity is even more pronounced in elite than in recreationally active individuals when comparing 485 and 3000 m (−18% vs. −12%, respectively) [31]. Moreover, the friction between SKIMO skins and the treadmill is different from that of the skins and the snow. Thus, environmental and technical-induced performance decrements have to be considered when laboratory results are transferred to the field. As an additional limitation, the small sample size of the present study needs to be mentioned. 

## 5. Conclusions

This study demonstrates that the applied SKIMO-equipment-specific exercise test is a valid assessment of $\dot{V}$O_2_max in recreationally active individuals. However, as no notable differences in HRmax or VTs were detected between protocols, the further benefit of the SKIMO-equipment-specific test must be questioned. Moreover, given the lack of comparison of the laboratory test results to SKIMO performance, larger-scaled studies, including field tests, are needed. Additionally, factors such as hypoxia and cold temperatures influencing aerobic capacity and, therefore, performance have to be considered when transferring the results to SKIMO training or competition. 

## Figures and Tables

**Table 1:** Demographic and anthropometric characteristics of the study group. Values are means ± SD.

Variables	Males (n = 11)	Females (n = 7)	Total (n = 18)
Age [years]	25 ± 3	26 ± 3	25 ± 3
Weight [kg]	78.1 ± 6.5	58.4 ± 4.8	70.4 ± 11.4
Height [cm]	182 ± 6	166 ± 7	176 ± 10
BMI [m^2^/kg]	23.5 ± 0.8	21.1 ± 0.9	22.6 ± 1.4
Exercise [h/week]	10 ± 7	10 ± 3	10 ± 5
SKIMO [tours/season]	38 ± 18	29 ± 8	34 ± 15

**Table 2:** Schematic of the uphill running protocol.

Time	Inclination (%)	Speed (km/h)
2	5	5.0
4	10	5.0
5	10	6.0
6	12	6.0
7	14	6.0
8	16	6.0
9	18	6.0
10	20	6.0
11	20	7.0
12	20	8.5
13	20	9.0
14	20	10.0
15	20	11.0
16	20	+1.0

**Table 3:** Schematic of the SKIMO protocol.

Time (min)	Inclination (%)	Speed (km/h)
2	10	3.0
4	20	3.0
5	20	3.5
6	22	3.5
7	24	3.5
8	26	3.5
9	28	3.5
10	30	3.5
11	30	4.0
12	30	4.5
13	30	5.0
14	30	5.5
15	30	6.0
16	30	+0.5

**Table 4:** Maximal and threshold values of the two exercise test protocols. Values are means ± SD.

Variables	Running	SKIMO	*p*-Value	Ƞ^2^p
$\dot{V}$O_2_max [mL/min/kg]	52.3 ± 5.8	52.3 ± 6.6	0.927	0.001
$\dot{V}$O_2_max [mL/min]	3692 ± 771	3710 ± 845	0.753	0.006
$\dot{V}$O_2_ VT1 [mL/min] $\dot{V}$O_2_ VT2 [mL/min]	2138 ± 569 3450 ± 715	2143 ± 534 3319 ± 747	0.953 0.148	0.000 0.119
HR VT1 [bpm]	135 ± 16	141 ± 18	0.083	0.167
HR VT2 [bpm]	181 ± 8	179 ± 11	0.431	0.037
HRmax [bpm]	192 ± 8	193 ± 9	0.587	0.018
BLAmax [mmol/L]	8.9 ± 1.9	7.7 ± 1.4	0.002	0.444
RERmax	1.18 ± 0.09	1.17 ± 0.08	0.960	0.000
RPEmax breathing	18.8 ± 1.1	18.8 ± 0.9	0.816	0.003
RPEmax legs	19.0 ± 0.8	18.3 ± 1.4	0.069	0.182
TTE [s]	729 ± 76	837 ± 74	<0.001	0.931

Ƞ^2^p, effect size partial ƞ squared; $\dot{V}$O_2_max, maximum oxygen consumption;$\dot{V}$O_2_ VT1, oxygen consumption at the first ventilatory threshold; HR VT1, heart rate at the first ventilatory threshold; $\dot{V}$O_2_ VT2, oxygen consumption at the second ventilatory threshold; HR VT2, heart rate at the second ventilatory threshold; HRmax, maximum heart rate; BLAmax, maximal blood lactate concentration; RERmax, maximum respiratory exchange ratio; RPEmax, maximum rating of perceived exertion; TTE, time to exhaustion.

## Data Availability

Data are contained within the article.
